# Strange Bedfellows: Coordinating Medicare and Medicaid to Achieve Cost-Effective Care for Patients with the Greatest Health Needs

**DOI:** 10.1007/s11606-020-05914-y

**Published:** 2020-05-27

**Authors:** Arielle Elmaleh-Sachs, Eric C. Schneider

**Affiliations:** 1 Department of Medicine, Massachusetts General Hospital, Boston, MA USA; 2 The Commonwealth Fund, New York, NY USA

## Abstract

This perspective describes federal efforts in the United States (U.S.) to integrate care for an especially complex, vulnerable, and costly patient population: adults eligible for both Medicare and Medicaid insurance. The goal of the paper is to demystify for clinical policy leaders and practicing clinicians the origins and evolution of the Dual-Eligible Special Needs Plans (D-SNPs) recently permanently authorized by the U.S. Congress and to explore the potential for these policy changes to help such health plans improve care for the sickest and most vulnerable Americans.

## DUAL ELIGIBILITY: PUBLIC INSURANCE FOR THE SICKEST AND MOST VULNERABLE AMERICANS

When Congress enacted Medicare and Medicaid insurance in 1965, it inadvertently created a bureaucratic challenge for Americans who straddle both programs, qualifying for Medicare because of age or disability and for Medicaid because of low income. These so-called dual-eligibles, now more than 12 million Americans, are among the poorest and sickest individuals in the USA.^1^ Typically, federal Medicare insurance covers acute care services, primary care, and prescription drugs, and state Medicaid insurance covers long-term services and supports, as well as Medicare premiums and cost sharing.^2^

Coverage by two distinct insurance programs adds to the risk that care will be fragmented and poorly coordinated. Policymakers have attempted to address this by modifying insurance benefits and payment approaches for the care of dual-eligible patients. In 2003, Congress introduced the Dual-Eligible Special Needs Plan (D-SNP) within Medicare Advantage to enhance coordination for this population.^3^ Since then, Congress made several additional adjustments to improve coordination and integration of services. In 2018, under the Bipartisan Budget Act, D-SNPs were permanently authorized.

A key question is whether actions by federal and state policymakers are enabling better coordination and less administrative burden for the dual-eligible population. This viewpoint article describes the structure of Medicare and Medicaid insurance and managed care plan offerings and examines their potential to integrate medical care and social services for this population.

Dual-eligible beneficiaries have a substantial mix of chronic medical and behavioral health conditions and disabilities and face significant social and financial challenges.^2^ They require a broad range of medical and social services. The population enrolled in both Medicare and Medicaid grew from 8.6 million in 2006 to 12.2 million in 2018.^1^ Approximately 39% qualified for Medicare benefits because of a disability.^1^ The number of beneficiaries under age of 65 grew faster than the number of those over 65. Of the dual-eligible population, 48% are from a minority race/ethnic group, 18% report poor health status, 43% do not have a high school diploma, and 55% have a least one limitation in activities of daily living.^1, 2^ Dual-eligible beneficiaries not only are more likely to report poorer health status but also account for disproportionately high spending for both Medicare and Medicaid programs.^2^

## MANAGED CARE TO ENHANCE COORDINATION: A PATCHWORK

**Fig. 1:**
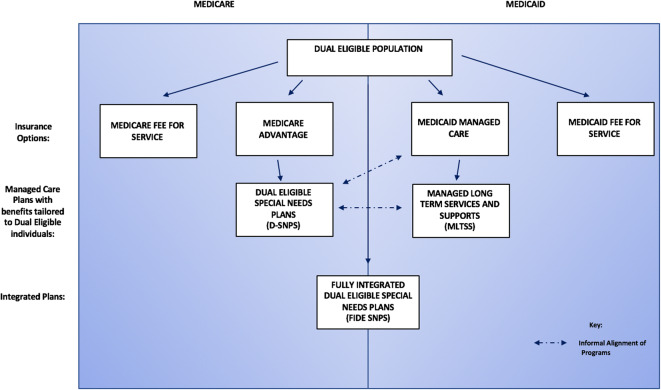
**Schematic of insurance options and plans available to Medicare and Medicaid dual-eligible beneficiaries**.

Medicare and Medicaid have turned increasingly to managed care to administer benefits for dual-eligible beneficiaries and enrollment has increased in both Medicare Advantage and Medicaid Managed Care plans.^2^ Nationally, most dual-eligible beneficiaries are in some type of managed care, although in some states fee-for-service arrangements remain prominent. Within managed care, several different plan options are offered to the dual-eligible population as described below. But the plans available to each beneficiary depend on the choices managed care organizations make available in each state and county (see Fig. 1).

Although the government has created three types of special needs plans for specific populations within Medicare Advantage (such as those with chronic conditions or individuals living in institutions), among dual-eligible beneficiaries, D-SNPs are the predominant type of plan, enrolling more than 20% of dual-eligible beneficiaries nationwide.^1, 4^ Table 1 outlines several approaches that have been tried to achieve coordination between D-SNPs and state Medicaid programs.^5^ Alignment between Medicare D-SNP and Medicaid plans varies markedly by state.

**Table 1:** Integration Options for Medicaid with Special Needs Plans for Dual-Eligible Medicare Beneficiaries

Type of alignment (from least to most integrated)	Description and requirements	Payment model	States and statistics
Dual-Eligible Special Needs Plans (D-SNPs)	- Must contract with state Medicaid agencies. - Subject to 8 minimum requirements to provide or arrange for Medicaid benefits, but integration not required.^7^	- Separate payments to Medicare and Medicaid.^6^ - No shared savings with Medicaid state programs.^6^	- 2.6 million enrollees. - 480 plans. - 42 states, PR and DC.^4^
D-SNPs with additional state-mandated covered services	States can require D-SNPs to:^3^ - Cover Medicare premiums and cost-sharing (typically paid by Medicaid). - Provide Medicaid acute care services not covered by Medicare (vision, dental, hearing, transportation, etc.). - Provide or arrange for Medicaid MLTSS or behavioral health services.	- If the D-SNP covers Medicaid benefits, and no companion Medicaid plan exists, Medicaid capitated payments may go directly to the D-SNP.^7^	- Varies by state.
D-SNPs with Medicaid Managed Long-Term Services and Supports (MLTSS) plans	- State Medicaid MLTSS plans either offer a companion D-SNP plan or states can require D-SNPs to operate MLTSS plans in the same geographic area.^7^ - Includes both Medicare and Medicaid benefits (LTSS and/or behavioral health services).^5^ - Allows for administrative alignment of these benefits when the plans are highly integrated.^5^	- If there is a companion Medicaid MLTSS plan, Medicaid capitated payments go directly to the Medicaid plan.^7^	-14 of 22 states with MLTSS programs have enhanced contracting between D-SNP and MLTSS.^3^
Fully Integrated Dual-Eligible Special Needs Plans (FIDE SNPs)	- Aligned Medicare and Medicaid benefits through a single managed care organization.^2^ - Integrated enrollment process and administrative alignment of Medicare and Medicaid.^3^ - Must cover Medicaid benefits with LTSS, and nursing facility services for at least 180 days per plan year.^9^	- Plan receives separate capitation payments from Medicare and Medicaid and integrates services.^5, 6^ - Savings from reduced Medicare service go to Medicare, no mechanism for shared savings with state.^6^	- 201,765 enrollees. - 45 plans. - 10 states.^4^

Medicaid covers services important to the dual-eligible population such as mental health benefits, non-emergency transportation, oral health, and primary care case management.^5^ The original 2003 legislation did not require D-SNP plans to contract with state Medicaid agencies, making it difficult for plans to coordinate an individual’s Medicaid-covered benefits. The Medicare Improvements for Patients and Providers Act (MIPPA) of 2008 required all D-SNPs to contract with states.^6^ D-SNPs could either include Medicaid benefits such as cost-sharing, or the acute services not covered by Medicare in their capitated package, or arrange for Medicaid benefits to be provided through a companion Medicaid plan.^3^

Many dual-eligible individuals have long-term care needs. Several states have embraced the Managed Long-Term Services and Supports (MLTSS) program—a capitated managed care alternative for long-term care.^6^ Dually eligible individuals in MLTSS have some of the highest per-beneficiary costs of all Medicare and Medicaid beneficiaries.^7^ With this in mind, state and federal policymakers have been trying to better align the administration and financial incentives of MLTSS and D-SNP plans.

## INTEGRATION: THE NEXT FRONTIER OR ANOTHER MIRAGE ON THE HORIZON?

As CMS and the states have seen limited progress with managed care, they have turned to solutions that try to bridge Medicare and Medicaid through integration. In addition to better care management, integration efforts strive to align incentives between Medicare and Medicaid in their payments and benefits along with the actions of plan administrators, care managers, and providers. Aligning incentives among these complex and independent entities is not a trivial undertaking.

To date, the main approach has been to consolidate payments in one budget managed by an accountable health plan. Fully Integrated Dual-Eligible Special Needs Plans (FIDE SNPs) were authorized in 2010 by the Affordable Care Act, but they are not yet widespread. These plans are designed to receive both Medicare and Medicaid capitation payments for the same beneficiary, combining them to create a single budget managed by a single-managed care organization, to administer and coordinate Medicare and Medicaid services for enrollees. Since 2013, when MIPPA went into effect, additional plan types have been introduced to integrate payment and delivery of services. They must also cover MLTSS services through risk-based Medicaid contracts.^7^ Plans receive a special designation from CMS and may qualify for a frailty adjustment payment based on their percentage of frail individuals.^3^ An additional attempt to align services for populations with specific needs is implemented by the so-called Highly Integrated D-SNPs (HIDE SNPs) that contract with the state to provide either LTSS or Medicaid behavioral health services.^8^

Efforts to align incentives between Medicare and Medicaid programs informed the Bipartisan Budget Act of 2018, in which Congress permanently authorized D-SNPs. Congress gave the CMS Medicare-Medicaid Coordination Office (MMCO) greater authority to assist in integration efforts and it unified beneficiary grievance and appeals processes for both programs.^2^ CMS recently established Medicare-Medicaid integration criteria for D-SNPs in 2021, where D-SNPs must either meet CMS requirements to be designated as a FIDE SNP *or* HIDE SNP, *or* a D-SNP must notify the state of hospital or skilled nursing facility admission for at least one high-risk population to allow for enhanced care coordination in transitions of care.^9^ The hope is that the plans will align Medicare and Medicaid services, generate cost savings per beneficiary, and improve health outcomes.

## ARE THESE PLANS AND MODELS IMPROVING QUALITY AND REDUCING COSTS?

Given the many new plan types and care models, it is not yet clear whether the actions taken by special needs plans are producing more integrated care for beneficiaries or whether plans are attracting enrollees. Whether new plans and care models are improving quality and health outcomes or producing cost savings remain open questions. Two models of care have been rolled out as demonstration and evaluation projects: the Financial Alignment Initiative and the Program of All-Inclusive Care for the Elderly (PACE). The Financial Alignment Initiative demonstrations received special authority from CMS to fully integrate Medicaid and Medicare benefits and payments in 13 states, either through a capitated model or a managed fee-for-service model, of which early results found some initial savings.^10^ Meanwhile, PACE integrates both Medicare and Medicaid benefits through an adult day health platform for the geriatric population who would otherwise be eligible for nursing home care. A review found no significant savings for Medicare, but an association with higher costs to Medicaid.^11^ While there were fewer inpatient hospitalizations for the PACE-enrolled individuals, there were higher rates of nursing home admissions.^11^

Similarly, there have been relatively few evaluations of the effects of D-SNPs on quality and health outcomes. One promising study that reviewed the Minnesota Senior Health Option, a care model administered by FIDE SNPs, found decreased hospitalizations and emergency department utilization.^3^ In Oregon, dual-eligible beneficiaries with aligned Medicare Advantage and Medicaid Managed Care plans experienced a reduction in hospital utilization and an increase in primary care visits, as well as greater likelihood of getting indicated screening tests for conditions like diabetes and high cholesterol.^12^

While increased D-SNP penetration has been associated in one study with reduced Medicare spending per dual-eligible beneficiary, state Medicaid budgets did not experience a similar benefit from these savings.^13^ Shared-savings could be used to motivate greater integration between Medicare and Medicaid.^3^ Given that FIDE SNPS are currently the only D-SNP plan that is financially at risk for all Medicare and Medicaid services, successful integration models developed by these plans should probably be adopted as the standard.^3^ Through bundled services under a single health plan and payer, there is greater incentive to control the costs for the population, and for organizations to benefit from any potential savings.^13^

## IMPLICATIONS FOR POLICY AND PRACTICE

Evaluations of current D-SNP and MLTSS-aligned models suggest several features integral to effective care coordination, including attention to transitions in care, information technology, data reporting and information sharing, caregiver participation, and a focus on social determinants of health.^2^ The specifications for models of care implemented by D-SNPs should be aligned across both Medicare Advantage and Medicaid care management requirements. Currently, care models are implemented separately by either Medicare Advantage and Medicaid, yet they overlap.^3^ Since care coordination is at the core of integrated plans, this would not only simplify the process for the beneficiary but also enable the models to work as intended.^3^ Further development and alignment of quality measures related to quality of life and care coordination needs of this population are needed to guide reporting and enable payment based on the D-SNP value proposition. At this time, the degree of integration between payments and the care delivered varies dramatically between states and between plans, making it difficult to discern the effectiveness of the various models. Alignment of D-SNPs with Medicaid MLTSS will require expanded geographic availability of D-SNPs and their provider networks, especially if an MLTSS program requires their Medicaid contractor to offer a D-SNP. Continued passive enrollment, or seamless conversion, of Medicaid-managed care enrollees to enter their companion D-SNP plan when they become eligible (i.e., age 65) will also help to broaden the availability of D-SNPs to beneficiaries.^3^

The commitment to special needs plans reflects a strong desire to reconcile potentially fragmented Medicare and Medicaid coverage and services for a vulnerable and costly population. Variation in D-SNP implementation may make evaluation challenging, but careful study may identify the features of these plans that enable some of the very sickest Americans to receive the highest quality health care at a price the nation can afford.
